# 519 Weekend Admission for Patients with Hidradenitis Suppurativa Have Better Outcomes than Weekday Admission

**DOI:** 10.1093/jbcr/irad045.116

**Published:** 2023-05-15

**Authors:** Rena Atayeva, Rafael Felix Tiongco, Carisa Cooney, Julie Caffrey, Jonathan Lee

**Affiliations:** Johns Hopkins University School of Medicine, Baltimore, Maryland; Johns Hopkins University School of Medicine, Baltimore, Maryland; Johns Hopkins University School of Medicine, Baltimore, Maryland; Johns Hopkins University School of Medicine, Baltimore, Maryland; Johns Hopkins University, Baltimore, Maryland; Johns Hopkins University School of Medicine, Baltimore, Maryland; Johns Hopkins University School of Medicine, Baltimore, Maryland; Johns Hopkins University School of Medicine, Baltimore, Maryland; Johns Hopkins University School of Medicine, Baltimore, Maryland; Johns Hopkins University, Baltimore, Maryland; Johns Hopkins University School of Medicine, Baltimore, Maryland; Johns Hopkins University School of Medicine, Baltimore, Maryland; Johns Hopkins University School of Medicine, Baltimore, Maryland; Johns Hopkins University School of Medicine, Baltimore, Maryland; Johns Hopkins University, Baltimore, Maryland; Johns Hopkins University School of Medicine, Baltimore, Maryland; Johns Hopkins University School of Medicine, Baltimore, Maryland; Johns Hopkins University School of Medicine, Baltimore, Maryland; Johns Hopkins University School of Medicine, Baltimore, Maryland; Johns Hopkins University, Baltimore, Maryland; Johns Hopkins University School of Medicine, Baltimore, Maryland; Johns Hopkins University School of Medicine, Baltimore, Maryland; Johns Hopkins University School of Medicine, Baltimore, Maryland; Johns Hopkins University School of Medicine, Baltimore, Maryland; Johns Hopkins University, Baltimore, Maryland

## Abstract

**Introduction:**

Hidradenitis suppurativa (HS) is a condition that involves recurring inflammation and fibrosis of intertriginous areas. With increasing inpatient hospitalization rates in patients with HS, our study sought to examine differences in outcomes between weekend and weekday admission. The authors hypothesized that patients admitted on a weekend will have worse in-hospital mortality rates when compared to those admitted on a weekday.

**Methods:**

We used a national database to perform a cross-sectional study focusing on 12,365 patients with a primary diagnosis of HS from 2017-2019. The variables measured were weekend or weekday admission, age, gender, race, and zip income quartiles, with the latter four identified as confounding factors controlled for in the logistic regression analysis. Descriptive analyses were performed to determine frequencies, means, and significance. Statistical significance was set at p< 0.05.The multivariate binomial logistic regression was used to calculate the odds ratio (OR) for the identified outcomes. The primary outcome was in-hospital mortality. The secondary outcomes were morbidity factors (infection and wound dehiscence rates, postoperative complications), time from admission to first procedure, and resource utilization (total hospitalization charges and costs, and length of hospitalization).

**Results:**

Weekend admission is not associated with worse inpatient mortality in patients with HS (*p*=0.345). From 2017-2019, 5 patients died, all of whom were admitted on a weekday. Compared to weekday, weekend admission had significantly lower incidences of wound dehiscence (0.38% vs 0%; *p*=0.007) and postoperative complications (0.24% vs 0%; *p*=0.035). No significant difference was observed for incidence of infection in weekday (0.43%) vs weekend admission (0.27%) (p=0.311). Weekend admission was also not found to have significant difference in the odds of contracting an infection (OR=0.74 (0.29-1.90); p=0.536). Weekend admission had significantly lower length of stay (mean days=6.40 vs 4.94; p=0.001), total charge ($64598.94 vs $44616.44; p< 0.001), and total cost ($14441.22 vs $10082.79; p< 0.001).

**Conclusions:**

In contrast to previous studies focused on other pathologies that have shown increased morbidity, treatment metrics, and resource utilization on weekend admission, our study demonstrates that specifically for patients with HS, weekend admission is associated with lower rates of morbidity, length of stay, and charge.

**Applicability of Research to Practice:**

Given that patients with HS who have a weekday admission have worse outcomes than a weekend admission, it is imperative for the medical team providing care to this group of patients on a weekday to be aware of this and begin to identify any potential factors.

